# Please help me, I am drowning! The cry of parents of adolescents with a substance use disorder

**DOI:** 10.4102/hsag.v29i0.2498

**Published:** 2024-02-16

**Authors:** Meriam M. Shadung, Paul R. Mbedzi, Rebecca M. Skhosana

**Affiliations:** 1 Department of Social Work, College of Human Sciences, University of South Africa, Pretoria, South Africa

**Keywords:** adolescence, parent, social work, substance use disorders, substance use

## Abstract

**Background:**

Substance use disorders (SUDs) are on the rise among adolescents worldwide, including in South Africa, causing a significant challenge to parents. Regardless of the difficulties associated with SUDs in adolescents and their impact on parents, current studies on substance addiction have focused on diverse fraternities, excluding social work services for parents of adolescents with SUDs.

**Aim:**

To develop an in-depth understanding of social work services provided to parents of adolescents with SUDs.

**Setting:**

The study was conducted in two districts, Capricorn and Waterberg, in Limpopo province, South Africa.

**Methods:**

A qualitative research method using exploratory and descriptive designs was employed. The scheduled interview guides facilitated semi-structured interviews with 11 social workers and 8 parents. For data analysis, Tesch’s eight steps were used.

**Results:**

Six themes emerged from the study: (1) social work interventions for parents, (2) family and community-related obstacles, (3) organisation-related obstacles, (4) substance use intervention strategies received by parents, (5) parents’ coping mechanisms in dealing with adolescents with SUDs and (6) factors hindering access to social services.

**Conclusion:**

The findings show that the government, particularly the Department of Social Development as the custodian of social services, is obliged to develop and design standard guidelines for services to parents of adolescents with SUDs and to provide uniform services to social workers.

**Contribution:**

The study benefits the social work profession, particularly in the field of substance abuse because it generates effective parameters for services for parents of adolescents with SUDs.

## Introduction

Substance use disorders (SUDs) among adolescents are increasing globally, including in South Africa, posing a huge challenge that also affects parents. Substance abuse by adolescents is a scourge affecting virtually all countries, including South Africa and Limpopo specifically, though the extent and characteristics vary (South African Department of Social Development (DSD) 2013). According to Mothibi (2014), SUDs in South Africa have ballooned to enormous proportions. It is a worrisome phenomenon in most South African villages and urban areas because youths die morally, socially, psychologically and physically (Lebese, Ramakuela & Maputle 2014). Similarly, Tshitangano and Tosin (2016) emphasise that substance abuse by adolescents in South Africa has reached critical levels, with the use of substances reported at twice the world average.

In South Africa, particularly in Gauteng, the South African Community Epidemiology Network on Drug Use (SACENDU) (2017) reports that 48% of clients who received SUD treatment at the centres were school referrals, indicating that it is adolescents who receive such treatment. Substance use disorders among South African adolescents present a challenge to society; a study reported that 13% of students in Mpumalanga and 25% in Limpopo were referred by schools to access treatment (SACENDU 2019).

Adolescent substance use does not only negatively affect users but also their parents. Mathibela and Skhosana (2019) demonstrate that professionals focus their concerns on adolescents abusing substances without attending to the vulnerability of their parents.

Anderson (2016) advises that the consequences of adolescents using substances do not only influence them but also affect their families, parents and society. Parents of adolescents with SUDs experience a flurry of emotions. Hoeck and Van Hal (2012) established that emotions that parents have to deal with range from worry to anxiety, helplessness and despair, depression and acute isolation. The authors add that the parents get worried about their child – not only about the drug abuse but also about the people they associate with. Such states of anxiety ultimately affect their physical and mental health, their financial insecurity, safety, education and work patterns. Most parents indicated that they experience increased stress that gets difficult to manage. Parents spoke of using strategies that, in retrospect, they observe as ‘crazy’ (Choate 2015).

One of the social worker’s roles is to regularly support individuals negatively affected by their own or someone else’s substance use (Galvani 2015). As recommended by Mathibela (2017), social workers need to develop suitable programmes to address the needs of parents with adolescents abusing chemical substances and their families. The author further recommends that such programmes should include parental support during the stages of treatment for adolescents.

Most interventions focus on parents as support structures for adolescents with SUDs during the treatment process but do not focus on the parent’s psychoemotional needs. Mathibela (2017:142) emphasises that ‘parents attend support groups to support the teenager abusing chemical substances’, whereas the focus should go beyond supporting adolescents with SUDs in maintaining sobriety to cater for the parents who must be assisted through support group sessions.

The aim of the study reported on was to develop an in-depth understanding of social work services provided to parents of adolescents with SUDs in Limpopo province. In the subsequent section, the problem statement is discussed in detail.

### Problem statement and purpose

Adolescent SUDs can have a negative influence on parents, particularly those experiencing lower socioeconomic status with increased difficulties in academic and social settings and family functioning (Choate 2015). In this regard, social workers must provide necessary support and services to parents of adolescents with SUDs (Galvani 2015). Mathibela and Skhosana (2019) established that social workers rather focus their attention on adolescents with SUDs than on the parents involved.

Most studies have focused on the experiences, challenges and coping strategies of parenting adolescents with SUDs (Choate 2015; Hoeck & Van Hal 2012; Kalam & Mthembu 2018; Masombuka 2013; Matheba 2020; Mathibela 2017; Mathibela & Skhosana 2019; Ngantweni 2018; Swartbooi 2013); however, no known study has explored the experiences of the social workers in rendering social work services to parents and the parents as the receivers of these services. It is, therefore, mandatory to identify and clarify the social work services for such parents as they find themselves in distressing situations. Hoeck and Van Hal (2012) indicate that social work services and support could alleviate some of the challenges that such parents encounter. In pursuit of such a trajectory, the purpose of the study was to develop an in-depth understanding of social work services provided to parents of adolescents with SUDs.

## Research methods and design

### Study designs and setting

A qualitative research method was used to describe and explore the social work services for parents of adolescents with SUDs in Limpopo province. Limpopo province is made up of five districts: Waterberg, Capricorn, Sekhukhune, Mopani and Vhembe. The study was conducted in two of the districts in Limpopo, namely, Waterberg and Capricorn.

### Population and sampling

Purposive sampling was employed in this study. In purposive sampling, the researcher determines what criteria are essential in choosing who is to be interviewed or what sites to observe (Sharan & Grenier 2019). The study sample consisted of 11 social workers with 2 years’ experience working in substance use, directly providing services and being able to communicate in English. The parent sample consisted of eight parents of adolescents with SUDs who have accessed social work services for the past 2 years and could communicate in any language in Limpopo province, e.g. Northern Sotho, Vhenda, Tsonga and English.

### Data collection

After permission was granted by the Limpopo DSD and SANCA Limpopo Alcohol and Drug Centre, contact was made telephonically concerning the date, venue and time for the interviews with participants. A total of 11 social workers and 8 parents participated in the study. Semi-structured, face-to-face interviews were used in conjunction with the interview schedule for data collection. As the country was under the COVID-19 lockdown restrictions, the researchers adhered to the COVID-19 regulations, ensuring that participants wore face masks, were sanitised and maintained social distancing.

### Data analysis

The data was transcribed by the researchers and the assistant of the independent coder was sought for data analysis. The researchers then enrolled an experienced independent coder to assist with data analysis. The independent coder analysed data using steps recommended and outlined by Creswell and Creswell (2018) to make sense of the collected data. The researchers consulted with the independent coder to get clarity on the identified themes.

### Measures of trustworthiness

Data were verified for the trustworthiness of the research findings by applying Lincoln and Guba’s model of credibility, transferability, dependability and conformability (Bryman 2012). Credibility was ensured by allowing the participants to review and check if what was recorded was a true reflection of what they stated during the interviews. Transferability was achieved by focusing on the selected sample that represented the population concerning the phenomenon. Dependability was ensured by enrolling an independent coder to code all transcribed interviews, and a report was generated for auditing by the supervisor to ensure that all necessary steps were followed. Conformability was through allowing the participants to express themselves fully without interruptions, more especially as the researchers once worked in the SUD field. The report documents the direct quotes as transcribed to support the findings and compare them with existing literature.

### Ethical considerations

The University of South Africa, College of Human Sciences Ethics Committee approved this study and issued an ethical clearance number (2020-CHS-34686622). Permission was granted by the Limpopo DSD research committee and SANCA Limpopo Alcohol and Drug Centre to conduct the study with the social workers rendering services in the SUDs field and parents of adolescents with SUDs. Participants were not exposed to any harm during the study; by showing acts of care, kindness and mercy were directed to the participants to avoid possible physical and emotional harm that could be caused other than being rude and inhumane. Further participants consented to their participation and were informed about their right to withdraw from the study at any given time. Confidentiality and anonymity of participants were ensured by keeping their names private and that the recording and transcripts were kept in a safe lockable place. Debriefing was made available for the participants who felt overwhelmed at the end of the interviews. The researchers avoided any possible physical and emotional harm.

## Results

There were 11 social workers, 7 males and 2 female participants. The social work participants were from young adulthood to adulthood, and ages ranged from 25 to 37 years, while one was 47 years. All the interviewees were black Africans from diverse ethnicities. The interviewed participants hold a 4-year social work qualification and are registered with the South African Council for Social Service Professions. With regards to parents, there were one male and seven female participants in the study. Participants’ ages ranged from 40 to 55 years, and one was 38 years. Most participants were black Africans with only one counterpart of colour. Most of the participants were primarily Sepedi-speaking, one was Tshivenda, one Tsonga and another Afrikaans. Of the seven female participants, one was married and six were single and the male participants were married. The findings from social workers and parents are presented separately and compared and contrasted in the discussion and conclusion.

## Themes and subthemes

Three themes and eight subthemes were identified from the social workers’ data: (1) social worker’s intervention provided for parents, (2) family and community-related obstacles and (3) organisational obstacles (see Table 1). Three themes and nine subthemes were also identified from the parents of adolescents with SUDs: (1) substance abuse intervention received, (2) parents’ coping mechanisms and (3) factors hindering access to services (see Table 2).

**TABLE 1:** Themes and subthemes arising from social workers.

Themes	Subthemes
1. Social work interventions for parents	1.1. Individual and group counselling 1.2. Parental education 1.3. Referral to relevant stakeholders
2. Family and community-related obstacles	2.1. Unavailability of parents 2.2. Financial constraints 2.3. Parent-social worker relationship
3. Organisation-related obstacles	3.1. Insufficient resources 3.2. Lack of support and supervision

**TABLE 2:** Themes and subthemes arising from the parents.

Themes	Subthemes
1. Substance use intervention received by parents	1.1. Exclusion from receiving services 1.2. Client assessment 1.3. Inpatient treatment admission 1.4. Casework/individual counselling
2. Parents’ coping mechanisms in dealing with adolescents with SUDs	2.1. Social support 2.2. Consultation with allied health professionals 2.3. Religious practices
3. Factors hindering access to services	3.1. Financial implications 3.2. Time constraints

SUDs, substance use disorders.

The themes identified by social workers are presented first, followed by themes identified by parents.

### Theme 1: Social work interventions for the parents

The social workers pointed out that they provide services mostly to parents identified as needing such services. They further indicated that there is no ‘one-size-fits-all’ prescription, meaning that parenting an adolescent with SUD does not automatically make the parent a candidate for their social services. In addition, they highlighted that they use social work tools such as assessment and screening to identify whether a parent is a suitable candidate for social work services. They also indicated that parents receive services based on the outcomes of the assessment, clarifying why some parents are not part of the intervention programmes offered.

#### Subtheme 1: Individual and group counselling

The social workers stated that they do consider the significant others of the adolescents with SUDs and engage them in the casework by providing individual counselling services to such parents. This subtheme is supported by the following vignettes:

‘In terms of the nature of social work services we provide for the parents of adolescents with substance use disorder, they form part of our treatment plan for the service users which are their children, being able to also render therapeutic individual counselling to the parents and educating them about substance use disorder. Also, [we] help them to acquire coping strategies as they are living with adolescents with substance use disorder.’ (Participant O, social worker)

‘Mostly we do casework more than group work, as it is challenging to bring these parents together, more especially if you find that some are employed, and they are not available during the week.’ (Participant R, social worker)

Similar to the brief reference to group work, other participants highlighted that their organisation does not focus on group counselling:

‘Okay, so far what I am doing is that I provide therapy in the form of individual sessions with parents. There are no group sessions currently that have been developed or provided to the parents of the adolescents. So, in these individual sessions one thing that I am trying to do is to counsel and educate them on how to cope with living with adolescents with substance use disorder, because in most cases you find that they don’t know what to do, they become … so we are also trying to provide emotional support to them, to encourage the issue of family support, so that they don’t give up on them.’ (Participant M, social worker)

‘Yes, one other thing that I should add is that currently, we don’t have a parenting group that is running in this office. What happens is that we provide individual counselling sessions to the parents, as there is no group.’ (Participant L, social worker)

#### Subtheme 2: Parental education

The participants were asked about the nature of services they provide to parents parenting adolescents with SUDs. A number submitted that parent education was offered to parents of adolescents with SUDs. The social workers offer education related to SUDs to the families so that they become knowledgeable about the disorder. Social workers educate parents on the substances, the consequences and how they could handle their adolescents on substances. This is important because such adolescents spend more time with their families than with social workers whom they only see on a consultative basis:

‘Remember these parents are supposed to be much aware of substance use disorders in relation to their kids that they are referring to us. So, they need to be well equipped, they need to be well informed about the substance use disorder that it is a brain relapsing disease, at the root level, and that is what we do conscientise them.’ (Participant Q, social worker)

‘Okay, thank you. So, with providing services to parents, we look at the impact of substances that the adolescent is using. If we look at the issues of finances, for example, that the adolescent has been abusing money from the family member or the parent, we are going to address that issue around finances. We educate them mostly about why and, okay, why would the adolescent steal money from them.’ (Participant K, social worker)

‘Then apart from that we also offer support services where we normally have sessions with the family either at our organisation or do home visits and at times, we educate them about this issue of substance use disorder because it is believed that this issue of substance use disorder is a family disease. Once it catches one person the entire family is affected.’ (Participant P, social worker)

#### Subtheme 3: Referral to relevant stakeholders

Still, on the question of the nature of social work services provided to parents parenting adolescents with SUDs, participants mentioned that they also do referrals based on the needs of their clients and families. Participant J stated that they assess the needs of the parents and refer them accordingly:

‘Not really, but if as a social worker, I identify that there are challenges that parents are facing not related to substance use disorders I refer to the relevant stakeholder.’ (Participant J, social worker)

‘And from there that’s when we will start to check what other available services can be provided for the family because it depends on the family per se. Not every family experiences the same thing from the same situation, yet all adolescents have a substance use disorder, but how they deal with that differs and the cause of substance use differs so we usually do that.’ (Participant F, social worker)

### Theme 2: Family and community-related obstacles

The participants were asked about the challenges they experience in working with parents of adolescents with SUDs, and they identified several barriers. There were three subthemes under this theme, namely family and community-related obstacles and organisational obstacles.

#### Subtheme 1: Unavailability of parents

It was verified that most parents of adolescents with SUDs are employed; therefore, it is difficult for social workers to engage them as part of their children’s treatment plan. This makes it also a challenge to create parent support groups because parents are unavailable during the week:

‘Eish … it is a struggle, it is difficult to reach the parents to access the services that we provide for them because most are employed and it is challenging to get each of them … The real challenge is getting parents together, like having a parent group, especially during the week as most of them have excuses that they cannot attend as they are at work during the day. That is the great challenge I come across in the field.’ (Participant J, social worker)

‘Another challenge here with regard to the parents … they are working. They are not able to attend the sessions … they just send someone to come and represent them and I just feel like it is different when it is the parent himself a herself there because you can assess how they respond to some of the things emotionally, psychologically.’ (Participant M, social worker)

On the other hand, parents come with preconceived ideas of what and how their children should behave despite having SUDs. This is further complicated by the fact that they hardly present themselves for the services with the social workers, making the interventions quite challenging:

‘They don’t avail themselves. Already they come with their expectations that they are not even ready to meet you halfway with whatever they have. They just believe that the adolescents should just listen to them and that’s it.’ (Participant O, social worker)

‘Yes, some just don’t want to be part and parcel of the treatment programme. They don’t show up for the scheduled appointment, it is more like they just want to drop off their children and they will come back to pick them up.’ (Participant J, social worker)

#### Subtheme 2: Financial constraints

Financial constraints hinder adequate access to social work services for adolescents with SUDs. The social work services rendered by some organisations are not free and not every parent can afford the consultation fees. The following quotes attest to the financial challenges experienced:

‘And the other challenge is money, that they cannot pay for their sessions as they are already paying for their children to access the services.’ (Participant J, social worker)

Participants M and N highlighted that lack of funds constrains parents from accessing social work services and that they become aware of the family’s financial challenges only during the assessment process:

‘And another challenge you find that some parents due to financial constraints wouldn’t be able to come to the session and you find out that once you miss that appointment with them, you will see them after a long period.’ (Participant M, social worker)

‘The parents don’t afford to pay for the consultation fees, and that one you pick it up when assessing that the family is not well doing financially and we work around that. Another challenge is money for transport to reach the office to get assistance.’ (Participant N, social worker)

#### Subtheme 3: Parent-social worker relationship

Parents of adolescents with SUDs tend to develop negative attitudes towards social workers, and this negatively disrupts the services rendered. This is depicted in the following storylines:

‘Honestly speaking the parents that are working, the ones that we declare professionals, are giving us a problem and they have these attitudes, they come here with the mentality of knowing it all, they’d even want to show you how to do the work until you start to sit down with them and plan well and engage with them how everything is going to unfold that is when they start to show up and some will respond well. Some will still maintain their attitude.’ (Participant Q, social worker)

The parents’ responses to social work services vary. Some parents have too high expectations of what social work services should do to change their children, forgetting that it is their responsibility, in collaboration with social workers, to transform the wayward behaviours of their children:

‘Yes, I can say with this one the response is different per parents. You get some other parents who have been struggling a lot with these adolescents who have substance use disorder, and they come to the office, and you find that the adolescent is still in the pre-contemplation phase and not ready to go through the programme and change. And the expectation tends to be high and with the knowledge of substance use the services that we render they expect immediate results because they are at your office.’ (Participant N, social worker)

‘The experience of working in this field of substance use disorder, especially looking at the parents of adolescents with substance use disorder, err … its, it’s kind of challenging because the parents … most of the time … they have expectations that this one is a child doesn’t have to say anything. It becomes hard if they don’t come to the issue of understanding and being part of the treatment.’ (Participant O, social worker)

### Theme 3: Organisational-related obstacles

#### Subtheme 1: Insufficient resources

The participants expressed their exasperation over the lack of the resources necessary for them to do their job. They argued that they are compelled to share computers and vehicles, making it difficult to reach out to the communities and their clients. The following sentiments were shared by the social workers:

‘About social work services, I would say the most common challenge that we do have is the issue of resources, looking at the time now you are interviewing me and the issue of the pandemic to reach out to other parents, and other communities.’ (Participant K, social worker)

‘We share computers in these offices. We share the vehicles, and that makes it difficult for us to visit the families of parents of children with substance use disorder.’ (Participant P, social worker)

#### Subtheme 2: Lack of support and supervision

Working with parents of adolescents with SUDs generates an emotional toll on the participants. The participants argued that they need to have self-control and emotionally detach themselves from their work because their experiences with clients can be heartbreaking as they cry, lash out and sometimes just feel hopeless:

‘We also don’t have enough support from the supervisors because they are forever out attending meetings, even when you come up across very new challenging cases because we experience new cases almost every time, so when you try to check for consultation and all those things it becomes a serious problem to us and that impedes the services that we want to render.’ (Participant Q, social worker)

‘There is no adequate supervision that is provided to us. That is why at times we have to provide services without proper guidance. I think that our supervisor is burdened as well, we draw up our plan for supervision sessions, but when it comes to adhering to it, our supervisor is always out. She is carrying so much on her plate, and that compromises the services we provide.’ (Participant C, social worker)

‘It needs uh … I don’t know … self-control, needs you sometimes to detach your emotions from what is happening because it is so heart-breaking as they come here and cry, they lash out sometimes they just feel like there is no need for help, so you need to show them that they need help and their adolescents need help. The parents themselves need help because they have been going through a lot. So you need to provide psychosocial and psychoeducational so that they understand what their adolescents are going through. And at the end, one needs support from supervision, which does not happen for a very long time.’ (Participant R, social worker)

Themes and subthemes emanating from parents’ data are presented as follows.

Table 2 shows the themes and subthemes that emerged from the data collected from parents.

### Theme 1: Substance use intervention received by parents

The participants were asked about the nature of services received from the social workers as the parents of adolescents with SUDs. Five subthemes emerged, namely, exclusion from receiving services, client assessment, inpatient treatment admission, case or individual counselling and family group session or conference.

#### Subtheme 1: Exclusion from receiving services

The parents were asked to identify the services they received, specifically meant for them as parents dealing with children with SUDs. The majority indicated that they had not received any services. Another challenge was that the services rendered by the social workers focused only on the substance user and there was little consideration of the parents and family members:

‘There is no service that I have received as a parent parenting a child with substance use disorder; only one family session was conducted with us.’ (Participant B, parent)

‘I am not sure if I must receive the services, as my child is receiving assistance there. There are no social work services that I as a parent have received from the Centre.’ (Participant D, parent)

‘I think the social workers are focusing much of their attention on our children, and yes because they are the primary clients and tend to forget about us as the parents or families.’ (Participant B, parent)

Participant E stated that the reason they came to the social worker’s office was because they felt burdened and wanted assistance. Unfortunately, the social worker only asked more questions about the person with the SUDs:

‘I think the social workers are focusing more on the affected person and they forget about us, that we are not coping and that at times we need support. I can tell that from the first time I bought my child here the focus was not on me and how I was affected by the whole condition because I visited the centre first without my son and I felt neglected.’ (Participant E, parent)

Participants A and B stated that they feel neglected as parents of children with SUDs. The participants affirm that they never received any social work services, only for their children. They further pointed out that the focus is on the person with SUDs and in their case, it is their children:

‘I don’t know if it’s due to workload or what, but as a parent of a child with substance use disorder I feel neglected and my other family members as well.’ (Participant B, parent)

‘No, I did not receive any social work service as a parent. It was all about my son referring him to the treatment and providing all the documentation needed to get him proper services. And as a parent of that child, I felt left out.’ (Participant A, parent)

#### Subtheme 2: Client assessment

The participants were asked about the nature of social work services they have received and they mentioned assessments. The assessments were defined as a process in which adolescents and their parents were seen individually by social workers to gather information on the substance user and help determine the intervention or treatment plan they needed. The process also involved completing documents:

‘My child went to access social work services voluntarily, and he was told to bring his parent to the next appointment. We went to the office we were seen by the social worker, who assessed us, where we were asked questions.’ (Participant B, parent)

‘I think I attended two sessions where the social worker was asking questions regarding the family background and other questions concerning my child.’ (Participant E, parent)

‘An assessment was conducted with me and my child by the social worker and thereafter my child was engaged in the intervention programme.’ (Participant G, parent)

Participant C indicated that after doing the assessments with the adolescents, they were placed on a treatment plan; the social worker also assessed with her to assist her:

‘That’s when she decided to have a formal assessment with me and we then agreed on the number of sessions and the focus areas, so that I can deal and cope with my challenges of parenting two children with substance use disorder. Yes, that is correct, it is just that I forgot how many sessions I had with the social worker.’ (Participant C, parent)

#### Subtheme 3: Inpatient treatment admission

Upon full assessments, some parents indicated that their children were then admitted into inpatient treatment centres:

‘After some time, we were informed that my son will be admitted. After admission at the in-patient Centre, we were not allowed to visit for three weeks, as he was still on detox.’ (Participant A, parent)

Participants A and B highlighted the process taken in referring the adolescents to the inpatient treatment centre. They indicated that after an assessment was completed with the social worker, they were referred to the hospital for a medical report to be completed:

‘We were then referred to the hospital to complete the medical report so that the application can be forwarded for in-patient treatment.’ (Participant A, parent)

‘And all the administration was completed, and my child was then referred to consult with the doctor so that the application can be completed for in-patient rehabilitation.’ (Participant B, parent)

Other participants specified that they had to follow up a bit more and inquire more with the social worker before their adolescent children were admitted for inpatient care as highlighted by Participant B as follows:

‘Yes, what I did is that I kept following up with the social worker on how long it would take for my child to be admitted because my son was troubling me, but finally I was called to prepare for him for admission.’ (Participant B, parent)

‘After three months from our first consultation, my son was sent to an in-patient rehabilitation Centre where he stayed for 3 months.’ (Participant D, parent)

#### Subtheme 4: Casework or individual counselling

It is quite normal that parents parenting adolescents with SUDs experience stress and other challenges. Orford et al. (2013) support the assertion that indeed parents of adolescents with SUDs often experience high levels of stress which significantly compromise their health and subjective well-being. As a result, they need someone to talk to and help them cope. Participant F stated that she received her counselling sessions from the social workers:

‘And I believe that the social worker arranged the sessions as he realised the condition that I was in, I was not coping at all. I did my sessions with the social worker, where I was tackling my challenges.’ (Participant F, parent)

Some of the participants indicated that the involvement of the social workers in the situation they had with their children assisted them in coping better. The individual sessions provided were helpful:

‘Okay, I had my challenges. I was not coping after learning that my son has a substance use disorder. I didn’t know how I assist him, and I was frustrated and angry. The involvement of the social workers helped me so much that I am even able to understand my son’s condition, and I now know how to be a supportive parent to him.’ (Participant H, parent)

‘I also had few individual sessions with the social worker where the main focus was on my mental wellbeing, like the stress I was experiencing due to my child using substances.’ (Participant G, parent).

### Theme 2: Parents’ coping mechanism in dealing with adolescents with substance use disorders

#### Subtheme 1: Social support

Some of the participants indicated that having a conversation with other people and support from friends and family were coping mechanisms that they used. Social support is also influenced by the parent’s desire to seek support, and for some, reluctance may result from self-blame, shame or fear of blame (Groenewald and Bhana 2018:650):

‘The support from my family and relatives was of utmost importance, they held me when I wanted to give up, they prayed with me and I was confident to open up to them about the challenges I was experiencing at that particular time.’ (Participant F, parent)

‘Women are talkative in nature, so I believe that talking with the other people assisted me to come to terms with my son’s condition. And now I feel okay and I can share with other people. I usually used to talk to the parents of the children that had the same disorder, and I also spoke to my family members because they provided support to me.’ (Participant A, parent)

#### Subtheme 2: Consultation with allied health professionals

Other participants indicated that they consulted with doctors, traditional healers and psychologists to try and cope with the challenges they experienced:

‘I do consult with the doctors and visit the clinic to get assistance in terms of the stress and high blood pressure that I experience as a result of the challenges brought by my son.’ (Participant B, parent)

‘When I was experiencing much stress, I used to visit my psychologist and even the doctor, as it was difficult for me to cope. And I would feel much better afterwards as the load would not be as heavy as it was. The doctor suggested that I see the psychologist so that we talk about the challenges that I have. And he was dealing with me on the medical part.’ (Participant E, parent)

‘I went to see my doctor several times and he was helpful in that I will get medication so that I will be able to sleep with peace. That is what I normally used to do before I received services from social workers. And to add I think the counselling worked better for me as I was allowed to express myself and how I felt at that moment than just taking medication only.’ (Participant H, parent)

The participants indicated that they usually rely on prescription medicine to cope with the challenges brought on by their adolescent’s condition. They claimed that they make use of both traditional and Western medications:

‘I once bought methadone for him to drink, but that didn’t assist as he relapsed after.’ (Participant A, parent)

‘I even tried traditional medicine in a bid to detox off the drugs to no avail. So, I will say I make use of the two.’ (Participant D, parent)

‘As an African, I had to consult with my traditional healer, and I felt better. You know at times when we encounter challenges in our families we need the intervention of a higher power, that is why I made such a consultation, and that is how some of us were raised. We need the guidance of our ancestors in all we do.’ (Participant C, parent)

#### Subtheme 3: Religious practices

Prayers and attending church are used as coping mechanisms for some parents in dealing with children with SUDs:

‘The other thing is that I also go to church and I think the women’s prayer sessions assist me in dealing with all these problems I am faced with, more especially a joint prayer where other fellow Christians pray with me. Exactly, that is the way that I use to help myself.’ (Participant B, parent)

‘I trusted that God will one day see me through the situation. I prayed and left it in God’s hands. And I also kept the hope that my child will one day better and stop using substances.’ (Participant I, parent)

On the other hand, participants indicated that they relied on and learned to pray because of their child’s condition. They find solace in their faith in God:

‘I relied more on God, I learned to pray more when I learned that my daughter has this condition and I would say that it worked for me, that I would all my heart out to my father in heaven.’ (Participant F, parent)

## Discussion

This study is intended to develop an in-depth understanding of social work services provided to parents of adolescents with SUDs. According to social workers, parents of adolescents with SUDs do receive services. Although the social workers indicated that they do provide individual and group counselling, parental education and support group facilitation, there is no consistency among the social workers, as some indicated that their organisation does not provide group work. The challenges encountered to access services result in difficulties in conducting group work.

The social workers’ responses confirm that there is a resource challenge in their departments, such as office space, vehicles and computers. Consequently, social workers noted that parents tend to become impatient when waiting to be seen due to a lack of offices. The social workers highlighted the fact that they must share vehicles, and Zastrow (2017) attests to the fact that often social workers must travel together to see clients and their families. Dhludhlu and Lombard (2017), Baloyi (2021) and Skobi (2016) concur that the lack of resources is a complicating reality as social workers were forced to drive in one vehicle and simultaneously conduct their respective home visits. This leaves them with little time to provide adequate services to their clients. Social workers are also expected to share one computer and submit approximately 10 reports per month.

The responses show that social workers must deal with high caseloads as they are expected to implement all nine social work programmes which lead to burnout. High caseloads lead to social workers feeling overextended, emotionally depleted and exhausted, affecting their quality of engagements with clients (DeLucia and Solano 2023). As the number of SUDs among adolescents increases, so does the need for social workers to meet the demand of service users. However, the higher caseloads result in social workers no longer being able to spend enough time with their clients due to their workload, which has a negative impact on the quality of work they deliver. Sekgobela (2021) cautions that the matter of quality work and the workload have placed a significant amount of pressure and responsibilities on social workers. Unegbu (2020) argues that the core barriers to the successful implementation of interventions on the part of social workers are compassion fatigue, burnout, countertransference and transference because they are attributed to job stress in substance abuse treatment centres. Social workers experience high levels of fatigue and cynicism linked to the organisation, client caseload, and these cumulatively compromise job performance.

Others argued that there is a great need for more social workers to fight the scourge of SUDs and to impact the communities. Bala and Kang’ethe (2021) recommend that the relevant authorities need to assign social workers to assist affected individuals, families and schools with counselling and prevention activities. The social workers must be engaged in the communities to effect change.

From the perspective of the parents, not all parents of adolescents with SUDs receive social work services, as most of the parents were perplexed when asked about the services received from social workers. The parents’ responses show that most parents of adolescents with SUDs are excluded from the social work services in Limpopo province. This is evident for the primary researcher (first author), as some participants found it difficult to distinguish between the services they received as parents and those provided to their children with SUDs. They were confused about which services were intended for them or their affected child. Some parents claimed that social workers were primarily concerned with the children with SUDs and completing inpatient treatment applications while neglecting them as parents. Hlahla, Ngoatle and Mothiba (2023) concur that most substance treatment centres treat people with SUDs, neglecting the families that brought them to the centre.

Both social workers and parents of adolescents with SUDs encountered challenges in providing and receiving services. The findings also show that working parents of adolescents with SUDs in Limpopo province are unable to access social work services. In a study conducted by Masombuka (2021), it was confirmed that the collapsed parent–child relationship and parents’ work commitment were among the main causes of parents’ unavailability and lack of cooperation in being involved in substance dependency for the youth. Mathibela (2017) argues that she observed a need for parents to take time off from work to attend support groups, as these groups are mostly facilitated by social workers during business hours.

The findings show that parents are unable to make time for social work services due to work commitments; additionally, parents claim that social workers do not provide clear schedules for the next appointment. Having to wait in long queues to access services compels them not to make time for social work services. This finding is consistent with Carpenter’s (2013) finding that it was difficult for employed parents to participate in the treatment of their substance-abusing child because their employment could be terminated if they took time to attend programme services. That is, it becomes difficult for them to attend the social work interventions that are intended for them. When parents do not make time for the interventions and do not offer support to the adolescents, it may be a contributor to their relapse (Mahlangu 2016). Some service users may interpret their parents’ absence as a sign that they have given up on them. Parents’ support is critical because failure to do so cannot help them break free from the current situation as articulated by Mahlangu and Geyer (2018).

The parents cannot travel to the social work offices because they are too far away; this places a financial burden on many because they have always had to pay for transportation to access the services. Some of them have to pay consultation or rehabilitation fees. According to Groenewald and Bhana (2018), rehabilitation costs include treatment costs as well as other expenses such as family costs associated with travel to and from treatment for visits and family meetings, as well as time away from work. Ngantweni (2018) confirms that parents experience financial loss while seeking or receiving treatment by having to pay for the intervention and transport. The social work services rendered by some organisations are not free; the parents must pay consultation fees for their children and themselves. As a result, money can be a barrier to a service user’s ability to succeed in their quest to become substance-free. When service users lack financial resources, they are compelled either to drop out of treatment or rely on state-funded treatment services, even though they must incur travel costs to access referring agents at outpatient sites (Baloyi 2021). Due to financial constraints, some parents can only afford to pay for their children’s services and not for their own, preventing them from accessing services that would assist them in coping with their difficulties (Shannon et al. 2015).

There is client resistance to working towards the objectives that are set by the social workers (Birkenmaier, Berg-Weger & Dewees 2011). The findings show that parents’ attitudes towards social workers and the services they provide differ from those of the parents with whom they work. This is supported by Ngubane (2021) in one study conducted in KwaZulu-Natal showing that parents may sometimes resist, reject the social worker and do not want a social worker involved in the family.

While some parents are harsh with the social workers, others respond patiently and understandingly, resulting in positive responses. This creates barriers to social work services aimed at parents of adolescents with SUDs, making it difficult for social workers to provide effective and efficient care. Ngubane (2021) further highlights that a lack of willingness to engage with the worker frustrates all efforts, even though the affected individual is likely to benefit from the mandated professional intervention.

## Limitations

The study focused on understanding the nature of social work services provided for the parents of adolescents with SUDs in Limpopo province from both the perspectives of the social workers and parents. It is important to note that Limpopo province consists of five districts, namely, Vhembe, Mopani, Capricorn, Sekhukhune and Waterberg. In this study, it became unfeasible for the researchers to include all the parents of adolescents with SUDs and social workers working in the substance use field around all districts of Limpopo province. Certainly, each district of Limpopo province has its own oddity and context which may influence the nature of social work services provided for the parents of adolescents with SUDs. Consequently, all parents of adolescents with SUDs and social workers working in the substance use field in Limpopo province cannot be accommodated in this study; the limitation, therefore, in this study is that only the social workers and parents of adolescents with SUDs from Capricorn and Waterberg districts participated. Though a limited number of social workers and parents were involved, the researchers managed to recognise and understand the nature of social work services provided for parents of adolescents with SUDs. For that reason, the findings emanated from this study cannot be generalised to be the reality in the entire Limpopo province.

## Conclusion and recommendations

The aim of the study was to develop an in-depth understanding of the nature of social work services provided to parents of adolescents with SUDs in Limpopo province. In conclusion, there are no standardised documented guidelines developed specifically for parents of adolescents with SUDs. The social workers rely more on the integrated service delivery method to provide services to parents, and it was also noted that the services provided are not uniform. The integrated service delivery method entails a framework for the incorporation of different programmes in the department, without being specific on the kind of service to be provided. A study inclusive of all districts of Limpopo province describing and exploring social work services to parents of adolescents with SUDs is recommended. All parents of adolescents with SUDs should go through screening, intake and assessment processes. More funding should be allocated to Non-Government Organisations (NGOs) providing SUD treatment to ensure that free services are extended to parents. Furthermore, DSD should develop a treatment programme specifically for parents of adolescents with SUDs. Employment of more social workers and supervisors in the substance use field ensures that there is manpower and that proper supervision is provided in the field. It is recommended that parents should be included in receiving social work services. There is a shortage of social workers who are knowledgeable about SUDs. The DSD and NGOs should recognise the critical need to return to basics by hiring more social work supervisors in the SUDs field, allowing for proper supervision of social workers. The findings have been supported by other studies and further elaborated and explained in the study reported on. However, the DSD has not responded to research by amending policies and frameworks and by providing logistical and financial support to render effective services.
